# COVID-19 associated vasculitis: A systematic review of case reports and case series

**DOI:** 10.1016/j.amsu.2022.103249

**Published:** 2022-01-13

**Authors:** Kalai Wong, Mir Umer Farooq Alam Shah, Maman Khurshid, Irfan Ullah, Muhammad Junaid Tahir, Zohaib Yousaf

**Affiliations:** a UNC Eshelman School of Pharmacy, USA; b Dow University of Health Sciences, Karachi, 74200, Pakistan; c Kabir Medical College, Gandhara University, Peshawar, Pakistan; d Lahore General Hospital, Lahore, 54000, Pakistan; e Department of Internal Medicine, Hamad Medical Corporation, Doha, Qatar

**Keywords:** IgA, Immunoglubulin A, SARS-C0V, Severe Acute Respiratory Syndrome Coronavirus 2, COVID-19, Coronavirus disease of 2019, COVID-19, Vasculitis, SARS-CoV-2, Kawasaki disease, Leukocytoclastic vasculitis, IgA vasculitis

## Abstract

Vasculitis is one of the complications of COVID-19. We conducted a systematic review analysing the association of COVID-19 with vasculitis. We searched Google Scholar and PubMed from December 1, 2019, to October 11, 2021. The review included 8 studies (7 case reports and 1 case series) reporting 9 cases of vasculitis secondary to COVID-19. The mean age was 29.17 ± 28.2 years, ranging from 6 months to 83 years. The male to female ratio was 4:5. Maculopapular, violaceous, papular and erythematous rash were common. Heparin(n = 2), corticosteroids (n = 6) (methylprednisolone) and intravenous immunoglobulin (n = 4) were prescribed in these patients. Significant clinical improvement was observed in 8 out of 9 patients. One person died during treatment. Our study discusses vasculitis as one of the complications of COVID-19. Furthermore, the pathophysiology, clinical presentation, and management of COVID-19 associated vasculitis is discussed.

## Highlights

• This is a systematic review of all published cases of vasculitis secondary to COVID-19 from the start of COVID-19 outbreak to date.
• Most common forms of vasculitis reported were Kawasaki disease Vasculitis, IgA vasculitis and Leucocytoclastic Vasculitis with symptoms being predominantly skin limited.
• Steroids, LMWH and IVIG were the most common line of treatment.
• An good overall prognosis was seen following treatment.

## 1 Introduction

With more than 241 million cases and 4.91 million deaths worldwide, coronavirus-2019 (COVID-19) has been a significant global economic and healthcare burden [1]. Several complications have been noted in patients of COVID-19. Vasculitis is the inflammation of blood vessels. It is triggered by autoimmune disorders, infections, and trauma [2]. There are different types of vasculitis, but leucocytoclastic (LCV), IgA, and Kawasaki disease like vasculitis are more commonly associated with COVID-19 patients.

IgA vasculitis (IgAV) is a systemic, immune complex-mediated, small-vessel vasculitis characterized by nonthrombocytopenic palpable purpura, arthritis, and abdominal pain. It typically occurs in children, although adults can also be a target of it. IgA vasculitis usually resolves spontaneously. Thus, only supportive treatment is advised [3]. Leukocytoclastic vasculitis, much like IgAV, is a small vessel vasculitis marked by immune complex-mediated damage. It appears as erythematous macules with palpable purpura appearing bilaterally on lower extremities and buttocks. However, unlike IgAV, it typically affects adults, although other groups are also at risk [4].

On the other hand, Kawasaki disease is a predominantly medium-vessel vasculitis that mainly affects children aged five years or younger. Patients typically present with fever, rash, and swollen hands and feet. If left untreated, it may cause heart complications and even death. Although reportedly globally, it has the highest incidence in Japan [5].

Medications commonly used to treat vasculitis include corticosteroids, immunosuppressive drugs, monoclonal antibodies, anticoagulants, anti-platelet agents, and immunoglobulin therapy. The choice of medications mainly depends on the type of vasculitis and the organ that is affected. As there is a rise in cases of COVID-19-associated vasculitis and vasculopathy, an effective treatment plan is yet to be discovered.

For over two years, varying complications of COVID-19 have been reported, and comprehensive systematic reviews of cases have come forth as a result [6,7]. However, the topic of vasculitis secondary to COVID-19 has been relatively untouched. To the best of our knowledge, this is the first systematic review of case reports and case series on the occurrence of vasculitis in COVID-19 patients. This article reviewed the relationship between COVID-19 and vasculitis, collated evidence, and summarized available literature.

## 2 Methodology

### 2.1 Literature review

Our work was conducted in line with the Preferred Reporting Items for Systematic Reviews and Meta-analyses(PRISMA) checklist (Supplementary file S1) [8]. We searched PubMed and Google scholar from December 1, 2019, to October 11, 2021, for studies published in English. Search terms were combined using appropriate Boolean operators. It included subject heading terms/keywords relevant to COVID-19 (e.g., SARS-CoV-2 OR Coronavirus Disease 2019 OR COVID-19 OR severe acute respiratory syndrome coronavirus 2 OR coronavirus infection) and vasculitis (e.g., Vasculitis OR Vasculitides OR Angiitis). The references of selected studies were also checked to ensure completeness of the search. Please see Supplementary File S1 for a sample search strategy. Furthermore, our study was registered in International prospective register of systematic reviews (PROSPERO) and holds the unique identifying number (UIN); CRD42021296389 [9].

### 2.2 Inclusion and exclusion criteria

Only case reports, case series, correspondence articles, and editorials that reported individual-level data on the emergence of vasculitis among COVID-19 positive patients were considered for this review. Studies were excluded if they did not present original empirical data on the clinical manifestation of the condition or reported aggregate-level data. The title, abstract, and full-text screening were completed in duplicate and independently by two reviewers (MUFAS and MK). Disagreements regarding the inclusion of studies for data extraction were resolved by the senior author (IU).

### 2.3 Data extraction

Duplicate studies were removed, whereafter data was extracted in case abstraction form on excel sheets. We collected data on age, gender, physical findings, type of vasculitis, treatment regimen, and treatment outcomes.

### 2.4 Quality assessment

We used the Joanna Briggs institute's critical appraisal tools to assess the quality of included papers [10,11]. Selected studies were examined for inclusion criteria, sample size, description of study participants and setting. Two reviewers independently assessed the methodological quality of each paper. Quality assessments were done with different tools based on different study designs. Each tool was modified to provide a numeric score. Tools had eight items for case reports and ten for case series. Included case reports (n = 7) had a mean score of 6.71 ± 0.45 with scores ranging from 6 to 7 [[12], [13], [14], [15], [16], [17], [18]]. Meanwhile, the case series by Akca et al. scored seven on a scale of 1–10 [19]. The detailed results of the quality assessment are provided in Supplementary file S1. The quality of our systematic review was assessed using AMSTAR 2 criteria [20]. The level of compliance with AMSTAR 2 came out to be “low”. As only case reports were included in the analyses and the number of studies were less, we couldn't conduct a meta-analysis.

## 3 Statistical analysis

We reported descriptive data of COVID-19 positive patients presenting with vasculitis. The data was presented in a table describing the symptoms, treatment, and subsequent prognosis among nine individuals who suffered from the condition(Table 1). The median and interquartile range (IQR) of the continuous variable (age) was calculated.

**Table 1:** **Patient information gathered from literature review of articles on individuals with vasculitis secondary to COVID-19.** (y/o = years old, m/o = months old, IV = intravenous, LMWH = Low molecular weight heparin, COVID-19 = Coronavirus disease 2019, VV-ECMO = veno-venous extracorporeal membrane oxygenation, IVIG = Intravenous immunoglobulin).

Author	Age and sex	Physical findings	Treatment	Diagnosis	Prognosis
Allez et al. [12]	24 y/o male	Skin rash, intense asymmetric arthralgia, periarticular swelling, and abdominal pain	LMWH, IV methylprednisolone (0.8 mg. kg)	COVID-19 associated IgA vasculitis	Discharged on day 7 under oral steroids and enoxaparin
Jones et al. [13]	6 m/o female	Erythematous, nonpruritic blotchy rash	Single-dose of 2 g/kg IVIG and high dose acetylsalicylic acid (20 mg/kg 4 times daily)	COVID-19 and Kawasaki Disease	Discharged on low-dose acetylsalicylic acid (3mg/kg daily)
Akca et al. [19]	7 y/o male	Diffuse erythematous maculopapular rash, erosive hyperemia of the oral mucosa	IVIG, azithromycin, hydroxychloroquine, ritonavir and lopinavir, tocilizumab, and mesenchymal stem cell treatments	Kawasaki-like disease and COVID-19	Died from severe hypoxia on the 17th day of VV-ECMO
Akca et al. [19]	10 y/o female	one-sided submandibular lymphadenopathy size of 2×1.5 cm, maculopapular erythema around the neck	IVIG, anakinra, corticosteroid therapies (20 mg/kg)	Kawasaki-like disease and COVID-19	Discharged on day 7
Sokolovsky et al. [14]	36 y/o female	Diffuse rash and arthralgias	Aspirin 650 mg, IVIG 2 g/kg, methylprednisolone 2 mg/kg	Kawasaki-like disease and COVID-19	Discharged home
Gómez et al. [15]	29 y/o male	Purple palpable papules	Corticosteroids (0.5 mg/kg/day)	Leucocytoclastic vasculitis and COVID-19	Skin lesions disappeared entirely after 15 days, and the patient remained asymptomatic after 3 weeks of follow-up
Hoskins et al. [16]	2 y/o male	Nonblanching, violaceous rash	LMWH, steroid	COVID-19 and IgA Vasculitis	Discharged home on a 4-week steroid taper, low-dose aspirin
Mayor- Ibarguren et al. [17]	83 y/o female	Purple palpable papules and sero-haematic blisters	Prednisone 30mg/day	Leukocytoclastic vasculitis secondary to COVID-19 infection	Clinical improvement after 10 days
Dominguez- Santas et al. [18]	71 y/o female	Pruritic macules and papules	Betamethasone dipropionate 0.05% cream twice daily.	Leukocytoclastic vasculitis secondary to COVID-19 infection	Lesions healed in the third week of follow up

## 4 Results

**Fig. 1:**
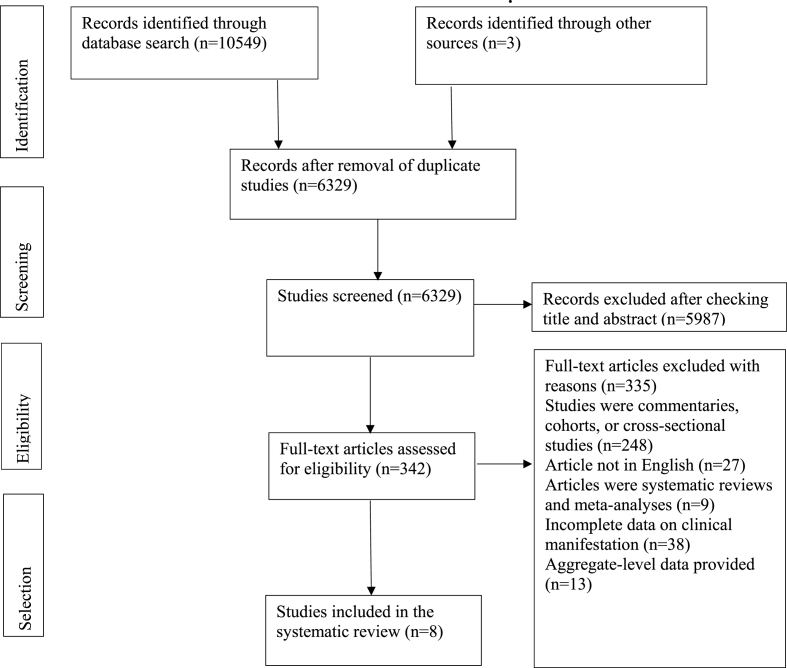
Flow chart of study selected for systematic review.

Our initial search yielded 10,552 results (Fig. 1). After removing duplicate studies (4223), titles and abstracts of the remaining studies were checked (6329). After excluding articles through title and abstract search (5987), full-text versions of the remaining articles were read (342). Studies were excluded if they were not in English (n = 27), reported aggregate-level data (n = 13), reported insufficient information on clinical events (n = 38), or were not of the desired study type (e.g., case report and case series) (n = 257). Finally, a total of 8 articles, comprising of 9 patients, met our inclusion criteria and were added to our qualitative analysis (Table 1).

Table 1 shows the information of 9 patients who developed vasculitis secondary to COVID-19. The median age of patients was 24 (49) years, ranging from 6 months to 83 years. The male to female ratio of patients was 4:5. Maculopapular, violaceous, papular, and erythematous rash were common presenting complaints. Other minor findings include arthralgia (n = 2), periarticular swelling (n = 1), lymphadenopathy (n = 1) and abdominal pain (n = 1). Although children did not complain of arthralgia, two adults presented with it. Common treatment regimen for patients with vasculitis secondary to COVID-19 included heparin(n = 2), corticosteroid therapy (n = 6) (methylprednisolone) and intravenous immunoglobulin (n = 4). Although a 7-year-old died of hypoxia, an overall good prognosis was observed (88.89%). Significant clinical improvement was observed in 8 out of 9 patients. 2 patients each were prescribed steroids and aspirin post-discharge.

## 5 Discussion

### 5.1 Pathophysiology

Endothelial inflammation, apoptosis, and dysfunction occur in patients with COVID-19 [21]. Endothelial cells are triggered by infection, oxidative stress, hypoxia, and environmental toxins. The external signals and intracellular mediators involved in this inflammatory property include anti-inflammatory cytokines, Transforming Growth Factor Beta (TGFβ), Interleukin 10 (IL-10), Interleukin 1 (IL-1) receptor agonist, and High Density Lipoprotein Cholesterol (HDL-C) [22]. Moreover, the widescale expression of Angiotensin Converting Enzyme 2 (ACE2) receptors within endothelial cells raises a question of its vulnerability to Severe Acute Respiratory Syndrome Coronavirus Disease 2 (SARS-CoV-2) binding, membrane fusion, and viral entry, causing infection, vascular injury, and dysfunction [21]. Accumulation of inflammatory cells and viral inclusions were identified within the endothelium. In autopsy and surgical tissue specimens, diffuse lymphocytic endotheliitis and apoptotic bodies were observed [23]. Thus, endotheliitis and endothelial cell injury can result in vasculitis amongst COVID-19 patients.

Kawasaki disease (KD) is an acute inflammatory disease characterized by medium-sized vasculitis with a predilection for coronary arteries, predominantly affecting children <5 years of age [24]. The leading theory for the pathogenesis of KD is that an unknown infectious agent leads to activation of the immune system in a genetically susceptible child, which can be supported by the apparent seasonality of KD [25]. It begins with neutrophil infiltration of the arterial wall, destroying the vascular wall and causing aneurysms. This phase is followed by lymphocytic infiltration with CD8+T cells, plasma cells, and monocytes, releasing pro-inflammatory cytokines Interleukin 1 Beta (IL-1β) and Tumor nNcrosis Factor Alpha (TNFα), which may continue for months to years in a few patients (chronic arteritis) [26,27]. As SARS-CoV-2 infection can accumulate inflammatory cells within the endothelium, endothelial inflammation, and dysfunction, it can trigger the development of KD. The systemic inflammatory response to pneumonia may potentiate the inflammatory response within coronary lesions, rendering endothelial dysfunction [23], resulting in the development of KD.

IgAV, formerly known as Henoch–Schönlein purpura (HSP), is a type of small-vessel vasculitis that is mediated by immune-complexes deposits containing IgA and is the most common form of systemic vasculitis in children [28]. The current etiopathogenetic hypothesis for IgAV assumes an abnormal immune response to various antigens in genetically susceptible individuals [29]. However, the exact etiology remains unknown. IgAV is usually preceded by upper respiratory tract infections, medications, vaccinations, or malignancies [30]. Thus, an upper respiratory tract infection by SARS-CoV-2 could be a triggering factor in the emergence of IgA vasculitis. It has been hypothesized that mucosal infections lead to upregulation of IL-6, which could lead to the development of Galactose Deficient IgA1 (Gd-IgA1) by altering glycosylation [31]. This variant of IgA1 antigenically recognizes structures of some microorganisms, anti-glycan IgA1 or Immunoglobulin G (IgG) antibodies, and forms circulating complexes, the deposition of these complexes can result in IgA vasculitis and nephritis. The cytokine storm and drugs are given during SARS-COV-2 infection could also be associated with COVID-19, resulting in IgA vasculitis.

Leukocytoclastic vasculitis (LCV) is a small vessel vasculitis characterized by immune complex-mediated inflammation of dermal capillaries and venules [4]. Although LCV is idiopathic in 50% of cases, it can also occur secondary to systemic autoimmune diseases, malignancies, drugs, and chronic infections. These factors induce immune complex deposition and complement-mediated damage within small vessels. Neutrophil chemotaxis followed by secondary exudation of fibrin, erythrocytes, and serum, injuring the vessel wall and causing fibrinoid necrosis. Elevated levels of serum IL-1, IL6, and IL-8 and tumor necrosis factors can also be detected [4]. Additionally, COVID-19 causes a cytokine storm linked with a rise in IL6 levels [16]. This rise in IL-6 levels and immune complex-mediated damage can result in inflammation of small vessels causing LSV.

### 5.2 Management

The treatment for COVID-19 induced vasculitis should be curated according to the severity of vasculitis, type of vasculitis, and patient demographics. The disease self resolves in most cases, however, based on the severity of skin-limited IgA vasculitis (sparing GI and renal involvement) a symptomatic treatment plan should be devised. Analgesics like acetaminophen should be prescribed for joint pain and muscle pain. Compression therapy and antihistamines should be prescribed for cutaneous variant of IgA vasculitis. This could reduce the formation of new lesions by inhibiting vascular dilatation [32]. Antihistamines work by inhibiting vasodilation, thereby, reducing blood supply. This prevents entry of immunoglobin to the affected area. Steroids should be given if there is a chance of incipient necrosis, which otherwise would slow down the healing process [32].

In KD, treatment comprises of high dose IVIG and aspirin [5]. Combinational therapy of IVIG, TNF inhibitors, steroids, calcineurin inhibitors, or anakinra, might be helpful to treat patients with IVIG-resistant disease [5].

LCV is mild and resolves with supportive measures like leg elevation, rest, compression stockings, and antihistamines. A 4–6 week tapering dose of corticosteroids can be used in more chronic or resistant cases. Rarely, immunosuppressive steroid-sparing agents such as methotrexate, azathioprine, mycophenolate mofetil, dapsone, cyclophosphamide, and intravenous immunoglobulin may be needed [4].

## 6 Limitations

This article has some limitations. For example, not all case reports included the patient's past medical history and risk factors for COVID-19 associated vasculitis, which may contribute to the new onset of rash. Some of the vasculitis cases are in asymptomatic COVID-19 patients, which raises the question about the correlation between COVID-19 and vasculitis. More cases were reported in children and adolescents, so more investigations are needed in diverse populations.

## 7 Conclusion

COVID-19 associated vasculitis is rare and lacks a clear treatment protocol. Steroid have shown clinical benefits in case reports. More studies are needed to establish a definitive treatment protocol for COVID-19 associated vasculitis.

## Ethical approval

Since this is a review article no ethical approval was needed.

## Sources of funding

The authors have no funding source to declare.

## Author contribution

Kalai Wong: study concept, study design, data collection, data analysis, data interpretation, writing the paper. Mir Umer Farooq Alam Shah: study concept, data collection, data analysis, writing the paper. Maman Khurshid: study concept, data collection, data analysis, writing the paper. Irfan Ullah: study concept, data analysis, writing the paper. Muhammad Junaid Tahir: study concept, data collection, data analysis, writing. Zohaib Yousaf: study concept, data analysis, writing.

## Consent

Not applicable since this is a review article.

## Registration of research studies

1. Name of the registry: PROSPERO
2. Unique Identifying number or registration ID: CRD42021296389
3. Hyperlink to your specific registration (must be publicly accessible and will be checked): https://www.crd.york.ac.uk/prospero/display_record.php?ID=CRD42021296389

## Guarantor

Kalai Wong, Maman Khurshid, Irfan Ullah.

## Provenance and peer review

Not commissioned, externally peer-reviewed.

## Declaration of competing interest

The authors have no conflict of interests to declare.
