# Supplementary material for: COVID-19 associated vasculitis: A systematic review of case reports and case series
Source: Ann Med Surg (Lond). 2022 Jan 13;74:103249. doi: 10.1016/j.amsu.2022.103249 (PMC8754903; doi:10.1016/j.amsu.2022.103249)
Supplement: Multimedia component 3 [file mmc3.docx]

Supplementary file

Search strategy for PubMed

1)COVID-19

"sars cov 2"[MeSH Terms] OR "sars cov 2"[All Fields] OR "sars cov 2"[All Fields] OR "covid 19"[MeSH Terms] OR "covid 19"[All Fields] OR "coronavirus disease 2019"[All Fields] OR "covid 19"[All Fields] OR "covid 19"[MeSH Terms] OR “covid 19 serotherapy"[All Fields] OR "covid 19 nucleic acid testing"[All Fields] OR "covid 19 nucleic acid testing"[MeSH Terms] OR "covid 19 serological testing"[All Fields] OR "covid 19 serological testing"[MeSH Terms] OR "covid 19 testing"[All Fields] OR "covid 19 testing"[MeSH Terms] OR "sars cov 2"[All Fields] OR "sars cov 2"[MeSH Terms] OR "severe acute respiratory syndrome coronavirus 2"[All Fields] OR "ncov"[All Fields] OR "2019 ncov"[All Fields] OR "coronavirus"[MeSH Terms] OR "coronavirus"[All Fields] OR "cov"[All Fields] OR "sars cov 2"[MeSH Terms] OR "sars cov 2"[All Fields] OR "severe acute respiratory syndrome coronavirus 2"[All Fields] OR "coronavirus infections"[MeSH Terms] OR ("coronavirus"[All Fields] AND "infections"[All Fields]) OR "coronavirus infections"[All Fields] OR ("coronavirus"[All Fields] AND "infection"[All Fields]) OR "coronavirus infection"[All Fields]

AND

2) Vasculitis OR Vasculitides OR Angiitis

"vasculitide"[All Fields] OR "vasculities"[All Fields] OR "vasculitis"[MeSH Terms] OR "vasculitis"[All Fields] OR "vasculitides"[All Fields] OR "angiitis"[All Fields]

3) Human

Filter

From 1^st^ December, 2019 to 11^th^ October, 2021

Search results:

699

Joanna Briggs Institute 2017 Critical Appraisal Checklist for Case Reports

| Author | Q1 | Q2 | Q3 | Q4 | Q5 | Q6 | Q7 | Q8 | Total |
| --- | --- | --- | --- | --- | --- | --- | --- | --- | --- |
| Allez et al.[] | yes | yes | Yes | yes | yes | yes | no | yes | 7/8 |
| Jones et al.[] | yes | yes | yes | yes | yes | no | no | yes | 6/8 |
| Sokolovsky et al. [] | yes | yes | yes | yes | yes | yes | no | yes | 7/8 |
| Gómez et al [] | no | yes | yes | yes | yes | yes | yes | yes | 7/8 |
| Hoskins et al. [] | yes | yes | yes | yes | no | yes | no | yes | 6/8 |
| Mayor-Ibarguren et al. [] | yes | yes | yes | yes | yes | yes | no | yes | 7/8 |
| Dominguez-Santas et al. [] | yes | no | yes | yes | yes | yes | yes | yes | 7/8 |

Joanna Briggs Institute 2017 Critical Appraisal Checklist for Case Series

| Author | Q1 | Q2 | Q3 | Q4 | Q5 | Q6 | Q7 | Q8 | Q9 | Q10 | Score |
| --- | --- | --- | --- | --- | --- | --- | --- | --- | --- | --- | --- |
| Akca et al. [] | Yes | yes | yes | no | no | no | yes | yes | yes | yes | 7/10 |

47/7
